# NSAIDs in the environment: a 2020–2025 review of impacts on plant and algal Physiology

**DOI:** 10.1007/s10646-026-03126-4

**Published:** 2026-07-29

**Authors:** Monika Majewska, Darya Harshkova, Anna Aksmann

**Affiliations:** Department of Plant Experimental Biology and Biotechnology, Faculty of Biology, University of Gdansk, Gdansk, Poland

**Keywords:** Non-steroidal anti-inflammatory drugs, Phytotoxicity, Photosynthesis, Respiration, Oxidative stress, Environmental contamination

## Abstract

Non-steroidal anti-inflammatory drugs (NSAIDs) belong to the most frequently detected pharmaceutical pollutants in aquatic ecosystems, raising growing concern about their effects on non-target primary producers. Unlike earlier reviews, based mainly on data collected before the year 2020, when environmental exposure levels were substantially lower, this work synthesizes research conducted over the last five years (2020–2025), a period that includes the SARS-CoV-2 pandemic. The pandemic was associated with a sharp global increase in the consumption of NSAIDs, resulting in their markedly elevated environmental loads. Consequently, the studies assessed in this review reflect plant and algal responses under significantly higher contamination pressures than those reported in pre-pandemic decades, offering a new perspective on their phytotoxic potential. A systematic literature search retrieved over 5,000 records, from which the most relevant experimental studies were selected for detailed evaluation. The compiled evidence demonstrates that NSAIDs adversely affect photosynthesis, induce ultrastructural damage to chloroplasts, and compromise mitochondrial respiration, including alterations in membrane potential and ATP production. Exposure to NSAIDs triggers oxidative stress responses, characterized by reactive oxygen species overproduction, lipid peroxidation, and variable changes in antioxidant enzyme activity. Beyond primary metabolism, numerous reports document disruptions in growth patterns, root system architecture, mineral balance, and secondary metabolite biosynthesis. By integrating the most up-to-date findings from a period of exceptionally intense pharmaceutical pollution, this review provides a novel and more realistic assessment of the ecological risks posed by NSAIDs. It underscores the urgency of developing stricter environmental quality standards and highlights key directions for future research under contemporary contamination scenarios.

## Introduction

Nonsteroidal anti-inflammatory drugs (NSAIDs) are widely used worldwide, leading to their frequent detection as contaminants in the aquatic environment (Nassri et al. 2023). By the late 1990s, the issue of environmental contamination by pharmaceutical substances and their potential threat to living organisms became the subject of intensive scientific research (Ternes 1998; Daughton and Ternes 1999). Over the years, interest in this topic has persisted, leading to the publication of numerous studies that have expanded knowledge of the subject. Studies conducted on invertebrates and vertebrates have shown altered detoxification enzyme activity, mitochondrial dysfunction, and reduced functional stability of cell membranes, impaired gene expression, and DNA damage (Tyumina et al. 2020). In higher plants and algae, phytotoxic effects have been manifested by, among others, growth inhibition, reduced photosynthetic pigment content, reduced photosystem II efficiency, inhibition of root elongation, reduced number and size of mature leaves, and induction of oxidative stress. These studies also highlighted the potential risk of NSAID accumulation in plant tissues (Bartrons and Peñuelas 2017; Alkimin et al. 2019; Sathishkumar et al. 2020; Madikizela et al. 2021). Over the past five years (2020–2025), attention to environmental contamination by NSAIDs has continued to increase. Moreover, the COVID-19 pandemic (2020–2023) significantly exacerbated NSAID contamination of the environment (Wojcieszyńska et al. 2022). Infection with SARS-CoV-2 (the virus responsible for COVID-19) was sometimes asymptomatic, but more commonly presented with inflammatory symptoms, including fever, dry cough, fatigue, and dyspnea, leading to elevated levels of inflammatory mediators such as C-reactive protein, D-dimer, and cytokines (IL-6, IL-8, TNF-α). Consequently, NSAIDs were frequently used both in clinical treatment and self-medication, resulting in increased pharmaceutical discharge into aquatic environments (Robb et al. 2020).

In literature, the most commonly studied NSAIDs are ibuprofen (IBU), ketoprofen (KET), diclofenac (DCF), and naproxen (NPX), all used worldwide (Krawczyk et al. 2023; Sha’aba et al. 2023). These pharmaceutical substances have been detected at various concentrations in environmental samples. For instance, KET concentrations in surface waters ranged from 14 to 190 µg L^− 1^, whereas in wastewater they ranged from 0.004 to 102 µg L^− 1^ (Krawczyk et al. 2023). Another example is DCF, which has been commonly detected in surface waters at concentrations ranging from 5 to > 72,000 µg L^− 1^ (Leverett et al. 2021).

Environmental risk assessment of NSAIDs employs multiple analytical approaches, with Species Sensitivity Distribution (SSD) representing a key statistical method in ecotoxicology. SSD is a widely used tool for assessing the varying sensitivities of different species to a given contaminant. This allows researchers to estimate the concentration at which a certain percentage of species within an ecosystem are expected to experience adverse effects. From the SSD analysis, the environmental quality standard (EQS) can be derived, representing safe concentration limits for a contaminant in aquatic ecosystems. These values serve as regulatory benchmarks to ensure environmental protection. In the DCF context, as a drug with relatively low biotransformation and metabolization by living organisms, the available chronic toxicity data in aquatic organisms are sufficient to establish EQS values (Leverett et al. 2021). Nevertheless, compliance assessments indicate that DCF concentrations in some European surface waters may exceed these proposed EQS values, highlighting potential environmental risks (Leverett et al. 2021). The European Commission has already proposed a new chronic EQS in 2022–2024: 0.05 µg L^− 1^ for diclofenac and 0.002 µg L^− 1^ for ibuprofen. Despite this, the implementation of these standards has been postponed until 2039, and the values are still subject to consultation (Council of the European Union 2024; Maack et al. 2022; Peters et al. 2022).

NSAIDs were initially designed for humans and animals, whereas their impact on “non-target” organisms, such as algae and plants, remains a crucial issue. As toxic substances, NSAIDs can affect plant cells through many of the mechanisms already mentioned (inducing oxidative stress, disrupting cell membrane integrity, and interfering with essential metabolic pathways). Drug toxicity depends on physicochemical properties (e.g., solubility, lipophilicity), concentration, and exposure time. Additionally, the plant’s characteristics, including its metabolism, anatomy, and physiology, play an important role, as species with more efficient detoxification systems may be less susceptible to these toxic effects. We decided to write this review to demonstrate that NSAIDs significantly disrupt photosynthesis and respiration in primary producers, suggesting the need for updated environmental quality standards and their rapid implementation in regulatory practice.

Reactive oxygen species (ROS) are most often mentioned in the context of a cell’s response to the toxic effects of substances. The organelles that act as both generators of ROS and producers of antioxidant enzymes in response to intoxication are chloroplasts, mitochondria, peroxisomes, and endoplasmic reticulum. ROS play a dual role in plants, both as signaling molecules and as toxic agents, which is why the reactions between organelles are closely linked (Wei et al. 2025). There is a wealth of literature on the interactions between chloroplasts, mitochondria, peroxisomes, and their functional connections with the endoplasmic reticulum (He et al. 2023; Molina-Moya et al. 2025; Rahikainen et al. 2025), including direct interactions via ROS. However, it remains challenging to determine which organelle is the primary source of the antioxidant enzymes produced in response to excessive ROS.

**Fig. 1:**
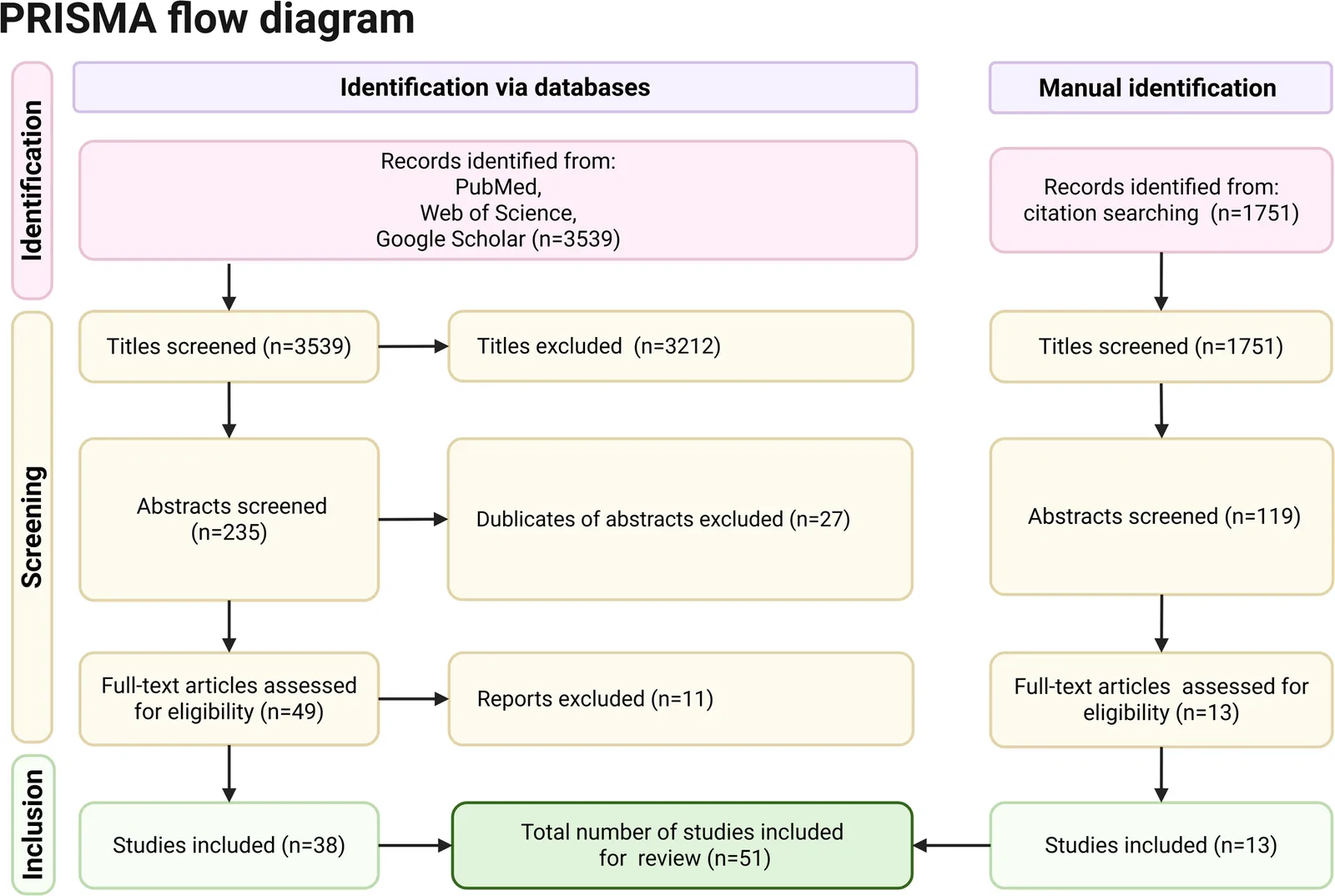
PRISMA-style flow diagram illustrating the literature search and study selection process for the review. Records were identified through searches of Web of Science, PubMed, and Google Scholar. Created in BioRender. Harshkova, D. (2026) https://BioRender.com/1ndl0li

Given the environmental persistence of NSAIDs and their widespread occurrence in aquatic ecosystems, understanding their impact on primary producers, the foundational organisms supporting all food webs, becomes critical for ecosystem health assessment. This review aims to analyze the current state of knowledge on NSAID-induced disruptions of fundamental physiological processes in algae and plants, specifically focusing on photosynthetic efficiency, cellular respiration dynamics, and associated pathological cellular alterations that may negatively impact entire ecosystems. To achieve this, a literature review was conducted covering all available publications from 2020 to 2025 on the impact of NSAIDs on plants and algae. Relevant studies were identified by searching the Web of Science, Google Scholar, and PubMed databases, using combinations of keywords including “NSAIDs”, “plants”, “algae”, “photosynthesis”, “respiration”, “oxidative stress”, “toxicity”, “growth”, “ultrastructure”, and “metabolism”. This strategy yielded approximately 5290 identified records. Publications not directly related to plant and algal physiology, or those focused exclusively on microbial degradation or wastewater treatment without biological effect endpoints, were excluded based on title and abstract analysis. Representative publications were selected according to the following criteria: original research articles published between 2020 and 2025, examining experimental studies focused on physiological, biochemical, metabolic, or ultrastructural effects of NSAIDs on algae or terrestrial/aquatic higher plants, without restrictions regarding tested concentrations, species, or individual drugs. All available papers meeting the above criteria regarding publication date, full-text availability, and thematic relevance were eligible for further analysis in this review. For each thematic section of the review, summary tables (Tables 1–4) were prepared, compiling all studies meeting the above criteria and systematically listing the target organisms (algae or plant species), the observed physiological, metabolic, and ultrastructural effects, the tested substances, and the references. Figure 1 illustrates the PRISMA-style flow diagram for the literature search and study selection process. The final version of the manuscript also included references added during the revision process to meet the Reviewers’ recommendations.

**Table 1:** Summary of recent reports (2020–2025) on the effects of non-steroidal anti-inflammatory drugs (NSAIDs) on photosynthesis in higher plants (A) and algae (B)

	Organism	Effects	Substances used for toxication	Year	Ref (DOI)
**A)**					
1	*Lemna minor*	no effect on the content of photosynthetic pigments	(Ketoprofen) KET	2020	Alkimin et al. (2020) https://doi.org/10.1016/j.envpol.2020.114993
2	*Solanum lycopersicum*	↑ chlorophylls content; ↑ expression of *D1* and *CP47* genes; ↓ expression of *rbcS* and *rbcL* genes; ↓ starch content	(Diclofenac) DCF	2020	Martins et al. (2020) https://doi.org/10.1007/s11356-020-09136-x
3	*Atriplex patula*	↓ chlorophyll *a* and *b*	(Naproxen) NPX, (Ibuprofen) IBU	2020	Opriș et al. (2020) https://doi.org/10.1016/j.plaphy.2020.03.046
4	*Spinacia oleracea*, *Lactuca sativa*	↓ chlorophyll *a* and *b*	NPX, IBU, DCF	2020	Opriș et al. (2020) https://doi.org/10.1016/j.plaphy.2020.03.046
5	*Lactuca sativa*	changing the shape and loosening the contents of the chloroplast	NPX	2020	Opriș et al. (2020) https://doi.org/10.1016/j.plaphy.2020.03.046
6	*Atriplex patula*	↑ amount of plastoglobules	NPX	2020	Opriș et al. (2020) https://doi.org/10.1016/j.plaphy.2020.03.046
7	*Spinacia oleracea*	chloroplast shrinkage	DCF, IBU	2020	Opriș et al. (2020) https://doi.org/10.1016/j.plaphy.2020.03.046
8	*Lactuca sativa*	chloroplast shrinkage	NPX, IBU	2020	Opriș et al. (2020) https://doi.org/10.1016/j.plaphy.2020.03.046
9	*Oryza sativa*	↑ PSII activity; ↓ electron transport efficiency; changes in expression of chlorophyll synthesis genes (↑ *HEMA* and *HEMG*; ↓ *CHLD*, *CHLM*, *CHLG*, and *CAO*); chloroplast swelling; ↑ starch content	KET	2020	Wang et al. (2020b) https://doi.org/10.1016/j.envpol.2020.114533
10	*Vigna unguiculata*	↓ chlorophyll *a*, *b*, and carotenoid content	IBU	2020	Wijaya et al. (2020) https://doi.org/10.3390/plants9111473
11	*Hordeum vulgare*	↓ chlorophyll *a*, *b*, and carotenoid content	DCF, NPX	2021	Pawłowska et al. (2021) https://doi.org/10.1016/j.etap.2021.103746
12	*Zea mays*	PS II activity disorders; ↓ chlorophyll *a*, *b*, and carotenoid content	DCF	2021	Siemieniuk et al. (2021) https://doi.org/10.3390/ijms22168856
13	*Zea mays*	↑ chlorophyll *a*, *b*, and carotenoid content; PS II activity disorders	NPX	2021	Siemieniuk et al. (2021) https://doi.org/10.3390/ijms22168856
14	*Solanum lycopersicum*	PS II activity disorders	DCF, NPX	2021	Siemieniuk et al. (2021) https://doi.org/10.3390/ijms22168856
15	*Hordeum vulgare*	↓ chlorophyll *a*, *b*, and carotenoids content;	ASA	2022	Biczak and Pawłowska, (2022) https://doi.org/10.1016/j.jenvman.2021.113936
16	*Cicer arietinum*, *Pisum sativum*, *Lens culinaris*, *Vicia faba*	↓ chlorophyll *a*, *b*, and β-carotene content; ↓ assimilation rates	DCF, indomethacin (IND), NPX,	2022	Taschina et al. (2022) https://doi.org/10.3390/app12136326
17	*Ocimum basilicum*	↓ chlorophyll *a*, *b*, and carotenoids content;	DCF, NPX, KET	2023	De Mastro et al. (2023) https://doi.org/10.3390/app13116759
18	*Hordeum vulgare*	↓ chlorophyll *a*, *b*, and carotenoids content;	IBU, KET	2023	Pawłowska et al. (2023) https://doi.org/10.3390/su15021613
19	*Lemna minor*	↓ chlorophyll *a*, *b* and carotenoids content; ↓ PSII activity; ↓ electron transport efficiency; ↑ RuBisCO amount	NPX	2023	Zezulka et al. (2023) https://doi.org/10.1016/j.aquatox.2023.106537
20	*Zea mays*	↓ chlorophyll *a*, *b*, and carotenoids content; ↓ PSII activity	IBU, DCF	2024	Biczak et al. (2024) https://doi.org/10.3390/su16135698
21	*Spinacia olerace*	↓ PSII activity; chloroplasts damage	DCF	2024	Majewska et al. (2024) https://doi.org/10.3390/plants13162189
22	*Chrysopogon zizanioides*, *Colocasia esculenta*	↓ chlorophyll *a* and *b* content	NPX	2024	Karki and Philip, (2024) https://doi.org/10.1016/j.cej.2024.149180
23	*Solanum lycopersicum*	↑ oxidized plastoquinone pull (qp); ↑ PSII activity; ↑ starch content	ASA	2025	Moustaka et al. (2025) https://doi.org/10.3390/ijms26031368
**B)**					
1	*Phaeodactylum tricornutum*	↓ electron transport efficiency; PSII activity disorders	IBU	2020	Silva et al. (2020) https://doi.org/10.1016/j.marenvres.2020.105109
2	*Scenedesmus obliquus*	PSII activity disorders; ↓ electron transport efficiency; chloroplasts damage, thylakoids membrane breakdown; ↓chlorophyll *a*, *b* and carotenoids content; ↓ expression of *psaA*, *psaB*, *psbB*, *psbD*, and *rbc*L genes	IBU	2020	Wang et al. (2020a) https://doi.org/10.1016/j.scitotenv.2019.136176
3	*Scenedesmus obliquus*	chloroplasts swelling, ↑ starch content, ↓ chlorophyll *a*, *b* and carotenoids content; PSII reaction centres damage; ↓ electron transport efficiency; ↓ oxygen evolution; ↓ expression of *psaA*, *ps*aB, *psbB*, *psbD*, and *rbc*L genes	KET	2020	Wang et al. (2020a) https://doi.org/10.1016/j.scitotenv.2019.136176
4	*Scenedesmus obliquus*	chloroplast deformations and disintegration; ↓ chlorophyll *a*, *b*, and carotenoids content; ↓ oxygen evolution, ↓ PSII activity; ↓ electron transport efficiency; ↓ expression of *psa*A, *psa*B, *psb*B, *psb*D, and *rbc*L genes	Acetylsalicylic acid (ASA)	2020	Wang et al. (2020a) https://doi.org/10.1016/j.scitotenv.2019.136176
5	*Chlorella* sp., *Desmodesmus spinosus*	↓ oxygen evolution; variable content of chlorophyll *a*, *b*, and carotenoids;	KET	2021	Gomaa et al. (2021) https://doi.org/10.1016/j.eti.2021.101586
6	*Chlorella* sp.	↓ oxygen evolution; ↑ chlorophyll *a*, *b*, and carotenoids content;	DCF	2021	Gomaa et al. (2021) https://doi.org/10.1016/j.eti.2021.101586
7	*Chlamydomonas reinhardtii*	↓ PSII activity; ↓ chlorophyll *a*, *b*, and carotenoids content; ↓ expression of *psaA* and *psbA* genes	DCF	2021	Majewska et al. (2021) https://doi.org/10.1016/j.ecoenv.2020.111630
8	*Chlamydomonas reinhardtii*	↑ starch content; ↓ chlorophyll and carotenoid content	IBU	2021	Moro et al. (2021) https://doi.org/10.1080/02757540.2021.1886279
9	*Mikraktynium simplicissimum, Desmodesmus* sp., *Chlorella pospolita, Lobosphaera* sp.	↓ chlorophyll *a*, *b*, and carotenoids content;	IBU	2022	Solovchenko et al. (2022) https://doi.org/10.1007/s10811-021-02660-4
10	*Desmodesmus* sp., *Chlorella pospolita*	↑ chlorophyll *a*, *b*, and carotenoids content;	DCF	2022	Solovchenko et al. (2022) https://doi.org/10.1007/s10811-021-02660-4
11	*Mikraktynium simplicissimum, Lobosphaera* sp.	↓ chlorophyll *a*, *b*, and carotenoids content;	DCF	2022	Solovchenko et al. (2022) https://doi.org/10.1007/s10811-021-02660-4
12	*Chlorella vulgaris*, *Desmodesmus armatus*	↓ chlorophyll *a* content	KET	2023	Krawczyk et al. (2023) https://doi.org/10.1016/j.scitotenv.2023.165019
13	*Chlorella sorokiniana*	↓ chlorophyll *a* content	IBU	2023	Sha’aba et al. (2023) https://doi.org/10.1007/s11356-022-22837-9
14	*Chlamydomonas reinhardtii*	↑ chlorophyll *a*, *b*, and carotenoids content; ↓ electron transport efficiency; ↑ oxygen evolution; ↑ starch content	Nabumetone (NBT)	2024	Kapuścińska et al. (2024) https://doi.org/10.1016/j.chemosphere.2023.140853
15	*Chlamydomonas reinhardtii*	↑ chlorophyll *a*, *b*, and carotenoids content; ↓ PSII activity; ↓ oxygen evolution; ↓ starch content	Flufenamic acid (FFA)	2024	Kapuścińska et al. (2024) https://doi.org/10.1016/j.chemosphere.2023.140853
16	*Chlorella vulgaris*	↓ photosynthetic activity	IBU	2024	Zhou et al. (2024) https://doi.org/10.1080/10889868.2022.2138823

↑ - an increase in parameter value was reported in the paper

↓ - a decrease in parameter value was reported in the paper

## Photosynthesis disorders as a result of NSAID toxicity

Photosynthesis, as a fundamental process underlying biomass production and oxygen release, is essential to ecosystem function, and its disruption can have significant ecological consequences for entire trophic networks. This chapter provides an overview of current knowledge on the effects of NSAIDs on photosynthetic processes in plants and algae. Notably, this area of research is still developing, and the exact mechanisms of NSAIDs action remain incompletely understood. Current studies suggest that some NSAIDs interact with the photosynthetic apparatus by altering the structure of chloroplasts, affecting the synthesis and stability of photosynthetic pigments and the efficiency of photosystem II (PSII), which is very sensitive to various types of stress (Murata et al. 2007). These disruptions can reduce photosynthetic efficiency, promote the production of ROS, and ultimately reduce plant vitality.

Diclofenac (DCF), one of the most widely used NSAIDs, is frequently detected in the environment. In recent years, multiple investigations have demonstrated DCF-induced disturbances in the photosynthetic efficiency of algal cells. Studies conducted by Majewska et al. (2021) on the unicellular green alga *Chlamydomonas reinhardtii*, exposed to DCF at a concentration of 134.0 mg L^− 1^, corresponding to the EC_50_ (value relative to the population density after 24 h), showed a few adverse effects on the photosynthetic apparatus. The observed changes included decreases in chlorophyll a, b, and carotenoids content, as well as a decline in PSII activity. These disruptions resulted in fewer active reaction centres, with some converting into non-photochemical energy dissipators, and, therefore, a decrease in the quantum efficiency of primary photochemical reactions (φP_0_) and the potential activity of PSII reaction centres (F_V_/F_0_). Moreover, DCF induced changes in gene expression, thereby causing a significant reduction in transcript levels of genes encoding PSII-*psbA* and PSI (photosystem I)-*psaA* core proteins. Reduced photosynthetic efficiency, as evidenced by decreased oxygen evolution in *Chlorella* sp. cells exposed to DCF at concentrations of 25, 50, and 125 mg L^− 1^, was also reported by Gomaa et al. (2021) and was found to be dose-dependent. However, chlorophyll and carotenoids content increased under these conditions. The differential impact of DCF on photosynthetic pigments content in green algae was examined by Solovchenko et al. (2022). In this study, four algal strains (*Micractinium simplicissimum*, *Lobosphaera* sp. IPPAS C-2047, *Desmodesmus* sp. IPPAS S-2014, and *Chlorella vulgaris*) were exposed to DCF at concentrations of 3, 300, and 1000 µg L^− 1^. At the highest concentration (1000 µg L^− 1^), a marked decrease in pigments content was observed in all strains except *Desmodesmus* sp. A similar, though less pronounced, reduction occurred at 300 µg L^− 1^ of DCF. Interestingly, the lowest tested concentration (3 µg L^− 1^) stimulated pigments accumulation in *M*. *simplicissimum*, *Desmodesmus sp*., and *C*. *vulgaris*, while no significant changes were detected in *Lobosphaera* sp.

In higher plants, DCF exposure has been shown to negatively affect photosynthetic pigment content and chloroplast structure. A marked decline in chlorophyll *a* and *b* content was observed in *Spinacia oleracea* and *Lactuca sativa* following DCF treatment at a relatively low concentration of 1 mg L^− 1^. In *S*. *oleracea*, DCF disrupted chloroplast morphology, leading to smaller, structurally compromised organelles (Opriș et al. 2020). In *Solanum lycopersicum*, exposure to DCF at concentrations of 0.5 and 5 mg L^− 1^ led to decreased chlorophyll content, increased carotenoids content, and reduced starch accumulation. Additionally, gene expression analysis revealed elevated expression of genes encoding PSII proteins (D1 and CP47) and reduced expression of genes involved in RuBisCO biosynthesis (*rbcS* and *rbcL*). Nevertheless, there were no significant changes in gas exchange (Martins et al. 2020). However, prolonged exposure of *S*. *lycopersicum* (7 and 14 days) to DCF (2 mg L^− 1^) resulted in significantly reduced photosynthetic performance, as evidenced by a decline in the maximum photochemical quantum efficiency of PSII (F_V_/F_M_) and the potential activity of PSII reaction centers (F_V_/F_0_). Additionally, a reduction in the content of photosynthetic pigments (chlorophyll *a* and *b*, and carotenoids) was observed (Siemieniuk et al. 2021). In contrast, *Zea mays* showed a much weaker response to DCF. Although PSII efficiency was slightly attenuated, the pigments content remained unchanged (Siemieniuk et al. 2021), suggesting a higher tolerance of *Z*. *mays* to DCF than *S*. *lycopersicum*. A dose-dependent reduction in chlorophyll and carotenoids content in *Z*. *mays* seedlings (1, 10, 100, and 1000 mg kg^− 1^ dry soil mass) was presented by Biczak et al. (2024), accompanied by a slight decrease in PSII activity at the highest concentrations. A significant loss of photosynthetic pigments was also demonstrated in *Hordeum vulgare* (Pawłowska et al. 2021), *Ocimum basilicum* (De Mastro et al. 2023), and several *Fabaceae* species (*Cicer arietinum*, *Pisum sativum*, *Lens culinaris*, and *Vicia faba*) after exposure to DCF. In the latter group, a DCF concentration of 0.5 mg L^− 1^ also reduced the CO_2_ assimilation rate (Taschina et al. 2022). Studies conducted on isolated chloroplasts and thylakoids from *S*. *oleracea*, treated with DCF in concentrations ranging from 125 to 4000 µM (Majewska et al. 2024), revealed progressive ultrastructural damage that intensified with increasing DCF levels. These changes were accompanied by inhibition of PSII activity, which is consistent with previous results (Majewska et al. 2021; Siemieniuk et al. 2021).

Biczak et al. (2024) assessed the effect of another commonly used NSAID, ibuprofen (IBU), on *Z*. *mays* seedlings. IBU caused a dose-dependent decrease in chlorophyll *a*, *b*, and carotenoids, as well as a slight reduction in PSII efficiency. Similar effects of IBU were observed in *Vigna unguiculata* (Wijaya et al. 2020) and *H*. *vulgare*. In the latter species, a slight increase in the chlorophyll *a* + *b* to carotenoid ratio was observed, indicating plant stress (Pawłowska et al. 2023). Opriș et al. (2020) showed that IBU at a concentration of 1 mg L^− 1^ caused a decrease in the content of chlorophyll *a*, *b*, and carotenoids in *Atriplex patula*, *S. oleracea*, and *Lactuca sativa*. Furthermore, it disturbed chloroplast morphology in *S*. *oleracea*, similar to what was observed for DCF.

In the context of unicellular algae, Wang et al. (2020a) conducted a comprehensive study on Scenedesmus obliquus exposed to IBU. The authors analysed pigments content, electron transport efficiency, chloroplast ultrastructure, and gene expression. Both racemic ibuprofen (rac-IBU) and its S-(+)-enantiomer were tested. Exposure to IBU resulted in severe chloroplast damage, including breakdown of the thylakoid membrane. Photosynthetic rate, chlorophylls, and carotenoids content decreased in a dose-dependent manner. Concurrent with the increase of IBU concentration, PSII damage was observed, as indicated by a gradual decrease in the values of the F_V_/F_M_, F_V_/F_0_, electron transport rate of PSII (ETR), and the primary light energy capture efficiency of the PSII reaction centres (Y(II). IBU treatment also decreased the relative expression of photosynthesis-related genes (*psaA*, *psaB*, *psbB*, *psbD*, and *rbcL*). Similar PSII disorders consisting of dysfunction of electron transport in the photosynthetic chain were observed in the diatom *Phaeodactylum tricornutum* exposed to IBU concentrations ranging from 0.8 to 300 µg L^− 1^, however, phytotoxic effects were only visible at the highest doses (Silva et al. 2020). Studies conducted by Moro et al. (2021) showed that *C*. *reinhardtii* exposed to IBU at concentrations of 62.5, 250, and 1000 µg L^− 1^ showed only a minor changes in the content of chlorophyll *a*, *b*, and carotenoids, and changes in chloroplast ultrastructure consisted mainly of intensive starch deposition. Solovchenko et al. (2022) investigated the response of four microalgae (*C*. *vulgaris*, *M*. *simplicissimum*, *Desmodesmus* sp. and *Lobosphaera* sp.) to IBU concentrations of 3, 300, and 1000 µg L^− 1^ and observed a concentration-dependent decrease in chlorophylls content and PSII photochemical efficiency, with *C*. *vulgaris* being the most sensitive. These results are consistent with those of Zhou et al. (2024), who also confirmed the inhibitory effect of IBU on the photosynthetic activity of *C. vulgaris*, with the effect increasing with increasing concentration. Interestingly, in a separate study on *C*. *sorokiniana*, Sha’aba et al. (2023) reported a significant increase in chlorophyll *a* content after 96 h of exposure to IBU at concentrations ranging from 10 to 10,000 µg L^− 1^.

Among the NSAIDs analysed by Opriș et al. (2020) on *A*. *patula*, *S*. *oleracea*, and *L*. *sativa*, naproxen (NPX) induced the most pronounced changes in chloroplast ultrastructure. In *L*. *sativa*, NPX exposure led to a change in shape and loosening content of the chloroplast. At the same time, in *A*. *patula*, a significant increase in the number of plastoglobuli was observed. Furthermore, all tested plants subjected to NPX exhibited a decrease in their photosynthetic pigment pool. A similar decrease in the content of chlorophyll and carotenoids due to exposure to NPX was observed in *H*. *vulgare* (Pawłowska et al. 2021) and only chlorophylls in *Chrysopogon zizanioides* and *Colocasia esculenta* (Karki and Philip 2024). However, the study by Siemieniuk et al. (2021) found that NPX at 2 mg L^− 1^ did not alter the content of chlorophyll a, b, and carotenoids in *S. lycopersicum* and even led to their accumulation in *Z*. *mays*. Nevertheless, both tested organisms showed reduced PSII activity, as evidenced by the declines in F_V_, F_M_, Area, F_V_/F_M,_ and F_V_/F_0_. Taschina et al. (2022) reported that NPX at a concentration of 0.5 mg L^− 1^ caused chlorophylls and carotenoids content loss and reduced CO₂ assimilation in four *Fabaceae* species (*C*. *arietinum*, *P*. *sativum*, *L*. *culinaris*, *V*. *faba*). It is worth noting that a comparable effect was also observed after exposure of these species to indomethacin (IND), which, to our knowledge, is the only report (in 2020–2025) on the effect of this NSAID on photosynthesis. A decrease in photosynthetic pigments content under the influence of NPX exposure was also observed in *O*. *basilicum* (De Mastro et al. 2023). In *Lemna minor*, exposure to NPX at 10 mg L^− 1^ contributed to a significant decrease in the content of chlorophylls *a*, *b*, and carotenoids, while the activity of PSII, namely F_0_ (fluorescence level at the initial stage when all PSII reaction centres are open), ΦII (effective quantum yield of PSII), F_V_/F_M_, and NPQ (non-photochemical quenching) were inhibited to a small extent. Interestingly, an increase in RuBisCO levels was observed (Zezulka et al. 2023). Notably, no studies reporting the effects of NPX on photosynthesis-related parameters in microalgae have been published in the last 5 years.

The next present in the environment NSAID with confirmed phytotoxic potential is ketoprofen (KET). As previously described, Wang et al. (2020a) investigated the response of the green alga *S*. *obliquus* to three NSAIDs–diclofenac (discussed earlier), acetylsalicylic acid (ASA, to be elaborated in the next section), and KET. Among the tested compounds, KET exhibited the strongest phytotoxicity. Observed effects included chloroplast deformation, evidenced by loosening of the thylakoid membranes, an increased number of starch grains, and the presence of osmophilic globules. With increasing KET concentration (0.01–6 mg L-1), a progressive decline in chlorophyll and carotenoids content was observed. Moreover, increasing doses of the drug deepened the damage to PSII reaction centres, as reflected by increases in F_0_ and F_M_ and decreases in F_V_/F_M_, Y(II), ETR, and qP (photochemical quenching). These changes corresponded with a decrease in the rate of photosynthetic oxygen evolution. KET at a concentration of 1 mg L^− 1^ also reduced the relative expression of photosynthesis-related genes (*psaA*, *psaB*, *psbB*, *psbD*, *rbcL*), similarly to IBU. In addition to *S*. *obliquus*, KET toxicity has been reported for two other microalgae, namely *Chlorella* sp. and *Desmodesmus spinosus*. In the article by Gomaa et al. (2021), a decrease in oxygen evolution rate was described, while chlorophyll content varied without a clear trend, and carotenoid levels increased. In the study by Krawczyk et al. (2023), a decrease in chlorophyll *a* content was observed. In higher plants, a KET-induced reduction in the content of chlorophylls and carotenoids in *Oryza sativa* seedlings was enhanced in a dose-dependent manner. Additionally, inhibition of PSII reaction centres activity and disruption of electron transport (decreased F_M_, F_V_/F_M_, F_V_/F_0_, and ETR parameters) were reported. At a concentration of 20 mg L^− 1^, KET induced changes in the expression of chlorophyll synthesis genes, increasing *HEMA* and *HEMG* expression, while decreasing *CHLD*, *CHLM*, *CHLG*, and *CAO*. Morphological alterations included swelling and increased starch accumulation (Wang et al. 2020b). A significant loss of chlorophylls and carotenoids was also noted in *H*. *vulgare* (Pawłowska et al. 2023) and *O*. *basilicum* (De Mastro et al. 2023). However, no change in the content of individual pigments was demonstrated after exposure of *L*. *minor* to KET (0.24, 1.2, 6, and 30 µg L^− 1^) (Alkimin et al. 2020).

Another NSAID worth mentioning is acetylsalicylic acid (ASA), whose precursor, salicylic acid, is naturally produced by several plant species, including *Salix* sp. and *Populus* sp. Although ASA is a commonly used NSAID, relatively few studies have examined its effects on plant cells. In *H*. *vulgare*, ASA caused a slight decrease in chlorophyll and carotenoids content, without altering the pigment ratio (Biczak and Pawłowska 2022). At the same time, *S*. *lycopersicum* treatment with ASA enhanced antioxidant protection and photoprotection, even under high-light conditions. It also improved PSII efficiency by increasing the fraction of open reaction centres and reducing the quantum efficiency of non-photochemical energy dissipation (NPQ). At the ultrastructural level, chloroplasts showed increased accumulation of starch grains (Moustaka et al. 2025).

Starch accumulation was also reported in the unicellular green alga *S*. *obliquus* (Wang et al. 2020a) treated with ASA at a concentration of 100 mg L^− 1^. However, in this case, treatment induced more severe photosynthetic dysfunctions, such as deformations and disintegrations of chloroplast, decline of chlorophyll *a*, *b*, and carotenoids, a photosynthetic rate reduction, PSII damage (increased F_0_ and F_M_, decreased F_V_/F_M_, and Y(II), and impairment of electron transport (decrease in ETR), and the observed effects intensified with increasing ASA concentration (10–150 mg L^− 1^). An ASA-induced decrease in the expression of genes related to photosynthesis (*psaA*, *psaB*, *psbB*, *psbD*, and *rbcL*), previously reported for IBU and KET, was also noted.

The accumulation of starch grains was observed as well in *C*. *reinhardtii* exposed to another NSAID, nabumetone (NBT), at 5.75 mg L^− 1^ (Kapuścińska et al. 2024), while no other significant changes in the chloroplast ultrastructure were observed. An increase in photosynthetic pigment content was observed, and despite a decrease in electron transport efficiency (φE0), a notable rise in oxygen evolution and water-splitting complex activity (FV/F0) was visible.

Another NSAID used in these studies, flufenamic acid (FFA) at a concentration of 35.47 mg L^− 1^, induced completely different effects in *C*. *reinhardtii* (Kapuścińska et al. 2024). Although FFA also led to the accumulation of photosynthetic pigments, to a lesser extent than NBT, it significantly reduced the pool of active PSII reaction centres, transforming some of them into dissipators of excess energy as heat (increased DI0/RC), resulting in a decrease in photosynthetic intensity. In contrast to NBT treatment, the cells’ starch content was reduced. Table 1 summarizes all reports on photosynthesis disorders caused by NSAIDs.

### Cellular respiration disturbances under the influence of NSAIDs

Although disruption of photosynthesis is the most conspicuous effect of NSAID toxicity, secondary effects on cellular respiration are equally crucial to understanding the complete physiological response of plants to pharmaceutical stress. Most studies on plants focus on photosynthesis, while the impact on mitochondrial metabolism and dark respiration is often overlooked. This is clearly evident in this review: there are far fewer studies on respiration than on photosynthesis. This section presents recent findings on how NSAIDs disturb respiratory activity and mitochondrial integrity in microalgae and higher plants. Reports regarding this part of the review are summarized in Table 2.

**Table 2:** Summary of recent reports (2020–2025) on the effects of non-steroidal anti-inflammatory drugs (NSAIDs) on cellular respiration in higher plants (A) and algae (B)

	Organism	Effects	Substances used for toxication	Year	Ref (DOI)
**A)**					
1	*Pisum sativum*	↑ membrane permeability	Naproxen (NPX)	2020	Svobodníková et al. (2020) https://doi.org/10.1016/j.chemosphere.2020.127411
2	*Oriza sativa*	damaged mitochondria	KET	2020	Wang et al. (2020b) https://doi.org/10.1016/j.envpol.2020.114533
3	*Atriplex patula*, *Spinacia oleracea*, *Lactuca sativa*	damaged mitochondria (ruptured membranes, SEM analysis), affected gas exchange	DCF, IBU, and NPX	2021	Opriș et al. (2021) https://doi.org/10.1007/s42729-021-00449-5
4	*Sauromatum senosum*, *Arum italicum*	mitochondrial energy imbalance	ASA, IBU, flurbiprofen, acetaminophen and DCF	2022	Skubatz (2022) https://doi.org/10.1111/plb.13466
5	*Pisum sativum*, *Lens culinaris*, *Vicia faba*, *and Cicer arietinum*	↓ gas exchange	DCF, indomethacin (IND), NPX	2022	Taschina et al. (2022) https://doi.org/10.3390/app12136326
6	*Oriza sativa*	↓ respiration, ↓ ATP, ↓ H⁺-ATPase activity	KET	2023	Wang et al. (2023) https://doi.org/10.1016/j.envpol.2023.122485
**B)**					
1	*Phaeodactylum tricornutum*	↑ mitochondrial electron transport	Ibuprofen (IBU)	2020	Silva et al. (2020) https://doi.org/10.1016/j.marenvres.2020.105109
2	*Scenedesmus obliquus*	↓ respiratory rates	IBU, acetylsalicylic acid (ASA), ketoprofen (KET)	2020	Wang et al. (2020a) https://doi.org/10.1016/j.scitotenv.2019.136176
3	*Chlamydomonas reinhardtii*	↓ MMP (mitochondrial membrane potential), changes in oxygen consumption	diclofenac (DCF)	2021	Harshkova et al. (2021) https://doi.org/10.1016/j.aquatox.2020.105698
4	*Chlamydomonas reinhardtii*	impact on oxygen consumption	Nabumetone (NBT)	2023	Kapuścińska et al. (2024) https://doi.org/10.1016/j.chemosphere.2023.140853
5	*Chlamydomonas reinhardtii*	impact on oxygen consumption, altered mitochondria structure	Flufenamic acid (FFA)	2023	Kapuścińska et al. (2024) https://doi.org/10.1016/j.chemosphere.2023.140853
6	*Chlamydomonas reinhardtii*	↓ MMP	IBU	2023	Seoane et al. (2023) https://doi.org/10.1016/j.aquatox.2023.106455
7	*Chlamydomonas reinhardtii*	↑ oxygen consumption, ↓ MMP, ↓ mtROS (mitochondrial reactive oxygen species), uncoupling of oxidative phosphorylation, mitochondrial deformations (elongation, irregular forms, degraded cristae, swelling/hyper-fission), transformation of vacuoles into larger autophagic vacuoles	DCF	2024	Harshkova et al. (2024) https://doi.org/10.7717/peerj.18005

↑ - an increase in parameter value was reported in the paper

↓ - a decrease in parameter value was reported in the paper

DCF is among the most studied NSAIDs concerning its environmental toxicity effects on respiration in algae and plants. In *C*. *reinhardtii*, exposure to DCF (135 mg L^− 1^) led to alterations in oxygen consumption, changes in mitochondrial membrane potential (MMP) depending on the exposure period, and morphological mitochondrial aberrations. Observed changes suggest impaired oxidative phosphorylation, supported by transmission electron micrographs showing elongated, irregularly shaped, or swollen mitochondria with degraded cristae, likely indicating mitochondrial swelling or hyperfission (Harshkova et al. 2021, 2024). In higher plants, DCF (0.1–1 mg L^− 1^) induces mitochondrial membrane damage, which has been demonstrated in *A*. *patula*, *S*. *oleracea*, and *L*. *sativa* (Opriș et al. 2021).

Impaired mitochondrial respiration has also been demonstrated following KET exposure. In *S*. *obliquus*, KET exposure (0.01, 0.1, 0, 1, 2, 6 mg L^− 1^) caused a dose-dependent decrease in respiration rate (Wang et al. 2020a). In *O*. *sativa* KET in concentration of 20 mg L^− 1^ induced mitochondrial atrophy and loss of mitochondrial structure (Wang et al. 2020b) and in concentration from 5 to 20 mg L^− 1^ reduced respiration rate, ATP levels, and H⁺-ATPase activity, while expression of H⁺-ATPase genes (*OSA9*, *OSA6*, *OSA10*) was upregulated (Wang et al. 2023).

Another drug proven to cause respiration disturbances is IBU. In the marine diatom *P*. *tricornutum*, increasing IBU concentrations (100–300 µg L^− 1^) resulted in increased mitochondrial electron transport activity (Silva et al. 2020), and in *C*. *reinhardtii*, IBU treatment (0.5–4 mg L^− 1^) led to a decrease in MMP (Seoane et al. 2023). As we mentioned above, when describing the operation of DCF, IBU (0.1–1 mg L^− 1^) in *A*. *patula*, *S*. *oleracea*, and *L*. *sativa* disrupts mitochondrial structures (Opriș et al. 2021). The work by Skubatz et al. (2022), which examined thermogenic processes in the floral appendages of *Sauromatum senosum* and *Arum italicum*, demonstrated the effect of IBU, DCF, and ASA on the activity of mitochondrial proteins such as alternative oxidase, F_1_F_O_-ATP synthase, and adenine nucleotide translocator, allowing the authors to clearly conclude that NSAIDs affect the energy balance of plant mitochondria (Skubatz et al. 2022).

In the case of other NSAIDs, NPX in concentrations from 0.1 to 1 mg L^− 1^ was shown to contribute to mitochondrial damage in *A*. *patula*, *S*. *oleracea*, and *L*. *sativa* (Opriș et al. 2021) and, in a concentration of 0.5 mg L^− 1,^ affected gas exchange in *Fabaceae* species (*C*. *arietinum*, *P*. *sativum*, *L*. *culinaris*, and *V*. *faba*) (Taschina et al. 2022). IND and flurbiprofen were linked to impaired thermogenesis in *S*. *senosum* and *A*. *italicum* through mitochondrial interference (Skubatz et al. 2022). NBT (5.75 mg L^− 1^) and FFA (35.47 mg L^− 1^) increased oxygen consumption in *C*. *reinhardtii*, and FFA was linked to structural disruption of mitochondria in this organism (Kapuścińska et al. 2024).

## Effects of NSAIDs on oxidative stress and antioxidant responses

Reactive oxygen species (ROS) accumulation and antioxidant defense mechanisms are fundamental components of the cellular response to anthropogenic pollutants, including pharmaceuticals (Hejna et al. 2022). In algae and plants, exposure to such contaminants often disrupts mitochondrial and chloroplast function, leading to an overproduction of ROS, including superoxide anions, hydrogen peroxide (H_2_O_2_), and hydroxyl radicals. These reactive molecules, while also playing roles in inter-organelle signaling, can cause oxidative damage. In response to stress, plant organisms activate a complex antioxidant network involving both enzymatic components (e.g., superoxide dismutase (SOD), catalase (CAT), and peroxidases (POX) and non-enzymatic molecules (e.g., glutathione, ascorbate, and phenolics). The balance between ROS generation and scavenging determines the extent of oxidative stress and is a critical marker of cellular toxicity (Wang et al. 2024). This section provides an overview of how different NSAIDs disturb redox homeostasis and trigger or suppress various antioxidative mechanisms.

Regarding algae, exposure of *C. reinhardtii* to 135 mg L^− 1^ DCF increased H₂O₂ production and CAT activity, without altering ascorbate peroxidase (APX) activity. Furthermore, there were changes in the expression of some genes associated with oxidative stress, namely increased *CAT1*, *MSD5*, *FDS1*, and decreased *APX1* (Harshkova et al. 2021; Majewska et al. 2021). In *Chlorella* sp. and *D*. *spinosus*, DCF exposure (25–250 mg L^− 1^) resulted in elevated malondialdehyde (MDA) levels and a decrease in CAT and APX activity, with more pronounced APX inhibition at lower concentrations (Gomaa et al. 2021).

In higher plants, *S*. *lycopersicum* cells exposed to two different DCF concentrations (0.5 mg L^− 1^ and 5 mg L^− 1^) showed changes in antioxidant enzyme activity, including a reduction in APX activity (Sousa et al. 2020), while glutathione S-transferase (GSTs) activity increased at 0.5 mg L^− 1^ and decreased at 5 mg L^− 1^ (Martins et al. 2020). In *L*. *minor*, DCF treatment at concentrations of 1, 2, 4, 8, 16, and 32 mg L^− 1^ resulted in a dose-dependent increase in CAT, GST, and glutathione reductase (GR) activities (Markovic et al. 2021). In *Z*. *mays* and *S*. *lycopersicum*, oxidative stress markers such as H₂O₂, MDA, and phenolic content increased under 2 mg L^− 1^ DCF (Siemieniuk et al. 2021). In *H*. *vulgare*, high concentrations of DCF (100–1,000 mg kg^− 1^ of solid growth medium) led to altered ascorbic acid content (Pawłowska et al. 2021).

KET is another often-mentioned NSAID in the context of oxidative stress response. In *Chlorella* sp. and *D*. *spinosus* cells, KET at concentrations ranging from 25 to 250 mg L^− 1^, similarly to the previously mentioned DCF, causes an increase in MDA content and a reduction in CAT and APX activity (Gomaa et al. 2021). *C*. *vulgaris* and *D*. *armatus* cells exposed to KET reacted by enhancing SOD, CAT, and POX activities (Krawczyk et al. 2023; Zhou et al. 2024). In *S*. *obliquus*, KET at concentrations of 0.01, 0.1, 0, 1, 2, and 6 mg L^− 1^ exposure caused a dose-dependent decrease in respiration rate and increased oxidative stress markers (MDA, O₂^•−^, H₂O₂, proline) (Wang et al. 2020a). In *L*. *minor*, KET (0.24–30 µg L^− 1^) stimulated GSTs activity (Alkimin et al. 2020), while in *O*. *sativa*, KET (20 mg L^− 1^) elevated expression of antioxidant enzymes, increased MDA and proline contents (Wang et al. 2020b).

Oxidative stress was also observed after IBU treatment (10, 50, 80, 100, 120, 150 mg L^− 1^) of *S*. *obliquus*. This consisted of elevated MDA, H₂O₂, and proline levels, as well as antioxidant-related genes induction (Wang et al. 2020a). In *P*. *tricornutum*, IBU (100–300 µg L^− 1^) stimulated antioxidant enzyme activity (CAT, APX, SOD) at lower concentrations, thereby mitigating oxidative stress (Silva et al. 2020). In *C*. *sorokiniana*, IBU exposure (10–10000 µg L^− 1^) induced H₂O₂ production, POX and GSTs activities, and lipid peroxidation (Sha’aba et al. 2023), while in *C*. *reinhardtii* IBU treatment (0.5–4 mg L^− 1^) caused ROS production (Seoane et al. 2023). In higher plant *V*. *unguiculata* (400–2000 ppm), IBU treatment in a concentration-dependent manner increased antioxidant enzyme activities (SOD, CAT, glutathione reductase (GR), and APX), and triggered oxidative stress responses (increased MDA and H₂O₂ levels) (Wijaya et al. 2020). In *H*. *vulgare*, IBU, as well as KET at concentrations of 0.1–1000 mg kg^− 1^ of dry weight of soil, changed ascorbic acid content, while IBU also caused an increase in SOD activity (Pawłowska et al. 2023).

ASA at concentrations of 10, 50, 80, 100, 120, and 150 mg L^− 1^ induced increased ROS production in *S. obliquus* similar to IBU and KET (Wang et al. 2020a). In *H*. *vulgare*, exposure to 0.1–1000 mg kg^− 1^ of solid growth medium ASA increased proline, MDA, and H₂O₂ levels (Biczak and Pawłowska 2022). However, foliar application of ASA at 1 mg mL^− 1^ in *S*. *lycopersicum* reduced ROS levels, indicating a context-dependent effect (Moustaka et al. 2025).

In *Z*. *mays* and *S*. *lycopersicum*, exposure to NPX (2 mg L^− 1^) increased H₂O₂, MDA, and phenolic content (Siemieniuk et al. 2021). In *H*. *vulgare* NPX, at concentrations of 0.1–1,000 mg kg^− 1^ of solid growth medium, altered ascorbic acid content and increased SOD activity were observed (Pawłowska et al. 2021). Oxidative stress induced by NPX (0.1–10 mg L^− 1^) in *P*. *sativum* was indicated by increased production of ROS, a higher MDA concentration, and variable CAT and APX activities–stimulating at low doses and inhibiting at high doses (Svobodníková et al. 2020).

Table 3 summarizes all reports on oxidative stress in plant and algal cells caused by NSAIDs.

**Table 3:** Summary of recent reports (2020–2025) on the effects of non-steroidal anti-inflammatory drugs (NSAIDs) on oxidative stress and antioxidant responses in higher plants (A) and algae (B)

	Organism	Effects	Substances used for toxication	Year	Ref (DOI)
**A)**					
1	*Lemna minor*	↑ GSTs (glutathione S-transferases) activity	Ketoprofen (KET)	2020	Alkimin et al. (2020) https://doi.org/10.1016/j.envpol.2020.114993
2	*Solanum lycopersicum*	↑ GSTs activity (dose-dependent)	Diclofenac (DCF)	2020	Martins et al. (2020) https://doi.org/10.1007/s11356-020-09136-x
3	*Solanum lycopersicum*	↓ APX activity	DCF	2020	Sousa et al. (2020) https://doi.org/10.1016/j.envpol.2019.113762
4	*Pisum sativum*	↑ MDA, ↑CAT and APX (ascorbate peroxidase) at low doses, ↓ CAT and APX at high doses, ↑ membrane permeability	Naproxen (NPX)	2020	Svobodníková et al. (2020) https://doi.org/10.1016/j.chemosphere.2020.127411
5	*Oriza sativa*	↑ MDA (malondialdehyde), O_2_^•^−and H_2_O_2_ , proline; ↑ expression of *FSD1*, *MSD1*, *CSD1*, *CSD2*, *CAT1*, *CAT2*, *CAT3*, *APX1*, *APX2*	KET	2020	Wang et al. (2020b) https://doi.org/10.1016/j.envpol.2020.114533
6	*Vigna unguiculata*	↑ SOD, CAT, APX, GR (glutathione reductase), ↑ H₂O₂, MDA	IBU	2020	Wijaya et al. (2020) https://doi.org/10.3390/plants9111473
7	*Lemna minor*	↑ CAT, GSTs, GR activity	DCF	2021	Markovic et al. (2021) https://doi.org/10.1016/j.ecoenv.2020.111428
8	Plants	Impact on phase I (cytochrome P450) and phase II (GSTs) drug-metabolizing enzymes	DCF, IBU, and NPX	2021	Mulkiewicz et al. (2021) https://doi.org/10.1016/j.scitotenv.2021.148251
9	*Hordeum vulgare*	↑ SOD (only NPX) activity, changes in ascorbic acid content	DCF, NPX	2021	Pawłowska et al. (2021) https://doi.org/10.1016/j.etap.2021.103746
10	*Zea mays*, *Solanum lycopersicum*	↑ H₂O₂, MDA, phenolic content	DCF, NPX	2021	Siemieniuk et al. (2021) https://doi.org/10.3390/ijms22168856
11	*Hordeum vulgare*	Altered proline, ↑ MDA, H₂O₂	ASA	2022	Biczak & Pawłowska, (2022) https://doi.org/10.1016/j.jenvman.2021.113936
12	*Hordeum vulgare*	↑ SOD (only IBU), changes in ascorbic acid content	IBU, KET	2023	Pawłowska et al. (2023) https://doi.org/10.3390/su15021613
13	*Chlorella sorokiniana*	↑ H₂O₂, POX, GSTs, lipid peroxidation	IBU	2023	Sha’aba et al. (2023) https://doi.org/10.1007/s11356-022-22837-9
14	*Solanum lycopersicum*	↓ ROS	ASA	2025	Moustaka et al. (2025) https://doi.org/10.3390/ijms26031368
**B)**					
1	*Phaeodactylum tricornutum*	altered antioxidant enzyme activity	Ibuprofen (IBU)	2020	Silva et al. (2020) https://doi.org/10.1016/j.marenvres.2020.105109
2	*Scenedesmus obliquus*	↑ MDA (malondialdehyde), O_2_^•^−and H_2_O_2_ , proline; ↑ expression of *FSD1*, *MSD1*, *CSD1*, *CSD2*, *CAT1*, *CAT2*, *CAT3*, *APX1*, *APX2*	IBU, acetylsalicylic acid (ASA), KET	2020	Wang et al. (2020a) https://doi.org/10.1016/j.scitotenv.2019.136176
3	*Chlorella sp*., *Desmodesmus spinosus*	↑ H₂O₂, MDA, CAT and APX activity	KET, DCF	2021	Gomaa et al. (2021) https://doi.org/10.1016/j.eti.2021.101586
4	*Chlamydomonas reinhardtii*	↑ CAT (catalase) activity, ↑ H₂O₂, ↑ expression of *CAT1*, *MSD5*, *FDS1*; ↓ expression of *APX1*	DCF	2021	Harshkova et al. (2021) https://doi.org/10.1016/j.aquatox.2020.105698
5	*Chlamydomonas reinhardtii*	↓ expression of *APX1*, and *MSD3* genes	DCF	2021	Majewska et al. (2021) https://doi.org/10.1016/j.ecoenv.2020.111630
6	Microalgae	Impact on phase I (cytochrome P450) and phase II (GSTs) drug-metabolizing enzymes	DCF, IBU, and NPX	2021	Mulkiewicz et al. (2021) https://doi.org/10.1016/j.scitotenv.2021.148251
7	*Chlorella vulgaris*	↑ SOD, CAT activity	KET	2022	Zhou et al. (2024) https://doi.org/10.1080/10889868.2022.2138823
8	*Chlorella vulgaris*, *Desmodesmus armatus*	↑ CAT, POX (peroxidase), SOD activity	KET	2023	Krawczyk et al. (2023) https://doi.org/10.1016/j.scitotenv.2023.165019
9	*Chlamydomonas reinhardtii*	↑ ROS	IBU	2023	Seoane et al. (2023) https://doi.org/10.1016/j.aquatox.2023.106455
10	*Chlorella sorokiniana*	↑ H₂O₂, POX, GSTs, lipid peroxidation	IBU	2023	Sha’aba et al. (2023) https://doi.org/10.1007/s11356-022-22837-9

↑ - an increase in parameter value was reported in the paper

↓ - a decrease in parameter value was reported in the paper

## Other manifestations of NSAIDs’ phytotoxicity

The previous chapters discussed the disruption of key metabolic processes induced by nonsteroidal anti-inflammatory drugs (NSAIDs). However, the effects of these xenobiotics extend beyond photosynthesis, respiration, and oxidative stress, representing either additional direct impacts or secondary changes resulting from primary metabolic disturbances. The summarizing of diverse morphological, anatomical, and physiological responses in algae and plants–from growth inhibition, through vacuolar reorganization and lipid accumulation, to disruptions in auxin transport and mineral metabolism is lighted in following chapter. Understanding these effects allows for a more comprehensive assessment of the toxicological potential of NSAIDs and the possible ecological consequences of their presence in the environment.

Several studies have investigated the effects of NSAIDs on the growth and development of algae and plants. Harshkova et al. (2021) demonstrated that DCF (135 mg L^− 1^) inhibited the growth of *C*. *reinhardtii* cells and caused cell cycle disruptions, likely due to the arrest of cell division. Further, KET (5–100 mg L^− 1^) significantly inhibited the growth of both *C*. *vulgaris* and *D*. *armatus* in a dose-dependent manner, with concentrations ≥ 50 mg L^− 1^ being lethal to cells (Krawczyk et al. 2023). In contrast to DCF and KET, IBU (62.5, 250, and 1000 µg L^− 1^) stimulated the growth of *C. reinhardtii* (Moro et al. 2021). Studies on higher plants have demonstrated multiple alterations in organisms exposed to NSAIDs. It was demonstrated that KET (0.5, 1, 5, 10, 20 mg L^− 1^) inhibited seedling growth in *O*. *sativa*, reducing their biomass (Wang et al. 2020b). The study by Svobodníková et al. (2020) revealed abnormalities in root anatomy, including shortened primary roots and reduced numbers of lateral roots, and reduced root and aboveground biomass in *P*. *sativum*. Significant impairments in root and shoot development were also observed in *Arabidopsis thaliana* treated with NPX (10–100 mM). These changes included shortening of primary roots, a reduction in the number of lateral roots, and impaired root and shoot gravitropism due to impaired auxin-dependent phototropism (Xia et al. 2023). Furthermore, *H*. *vulgare* treated with DCF and NPX (0.1–1000 mg kg^− 1^) initially showed increased root length, but after the highest concentrations, a significant reduction in root length was observed (Pawłowska et al. 2021). Moreover, NPX exposure (0.1, 1, 10 mg L^− 1^) inhibited root elongation in *L*. *sativa*, *P*. *sativum*, and *A*. *porrum*, whereas *Z*. *mays* treated with DCF or NPX at corresponding concentrations exhibited root elongation (Kummerová et al. 2024). NSAIDs exposure was also reported to induce changes in cellular ultrastructure. After exposure of *C*. *reinhardtii* to NBT (5.75 mg L^− 1^), FFA (35.47 mg L^− 1^), and DCF (135 mg L^− 1^), vacuoles transformed into large autophagic vacuoles (Harshkova et al. 2024; Kapuścińska et al. 2024). After exposure to IBU, lipid and protein content increased in *P*. *tricornutum* (Silva et al. 2020) and C. *sorokiniana* (Sha’aba et al. 2023) cells. In *C*. *reinhardtii* treated with IBU (62.5, 250, and 1000 µg L^− 1^), numerous electron-dense inclusions were observed in vacuoles, and with increasing IBU concentration, lipid accumulation in the cytoplasm increased (Moro et al. 2021). Lipid accumulation was also observed in the cells of terrestrial plants. Oil accumulation in cell walls and vacuoles after exposure to DCF, NPX, and IND was demonstrated in *A*. *patula*, *S*. *oleracea*, and *L*. *sativa* (Opriș et al. 2021), while in *P*. *sativus*, after the application of NPX (0.1–10 mg L^− 1^), the opposite effect was observed, i.e., a decrease in the total protein content in roots (Svobodníková et al. 2020). These studies also demonstrated damage to the cell membrane, resulting in a change in its permeability. The same effect was demonstrated in *O*. *sativa* after exposure to KET (0.5–20 mg L^− 1^) (Wang et al. 2023) and *C*. *vulgaris* and *D*. *armatus* (5–100 mg L^− 1^) (Krawczyk et al. 2023). Membrane depolarization was also demonstrated in *C*. *reinhardtii* exposed to IBU (0.5–4 mg L^− 1^) (Seoane et al. 2023).

Alterations in stomatal function induced by NSAID exposure have also been reported, and in *A*. *patula*, *S*. *oleracea*, and *L*. *sativa*, DCF, NPX, and IND (0.1–1 mg L^− 1^) caused changes in stomatal index and pore length, with the most significant changes observed in plants treated with NPX (Opriș et al. 2021). The same substances (in 0.5 mg L^− 1^ concentration) reduced stomatal conductance in *C*. *arietinum*, *P*. *sativum*, *L*. *culinaris*, and *V*. *faba* (Taschina et al. 2022).

Mineral homeostasis disruption was another problem reported in plants treated by NSAIDs. In *O*. *sativa* KET (1–20 mg L^− 1^) exposure caused disturbances in nitrogen metabolism, consisting of reduced the content of nitrates, ammonium ions, and amino acids, as well as the activity of key nitrogen metabolism enzymes (e.g., nitrate reductase, nitrite reductase, glutamine synthetase) (Wang et al. 2023). In *V*. *unguiculata* (400–2000 ppm), IBU treatment altered mineral uptake in a concentration-dependent manner, reducing potassium and magnesium content and increasing calcium and manganese content (Wijaya et al. 2020).

NSAIDs demonstrated significant impacts on plant secondary metabolism and volatile emissions. Taschina et al. (2022) observed a decrease in flavonoids concentration and an increase in 1-hexanol emission by *C*. *arietinum*, *P*. *sativum*, *L*. *culinaris*, and *V*. *faba* after exposure to DCF, IND, and NPX (0.5 mg L^− 1^), with a decrease in phenol concentration in *L*. *culinaris*. However, in *O*. *sativa* seedlings, the content of 2,3-butanediol and ethanolamine significantly increased at a KET concentration of 20 mg L^− 1^ (Wang et al. 2020b). Reports summarizing this part of the review are presented in Table 4.

**Table 4:** Summary of recent reports (2020–2025) on the effects of non-steroidal anti-inflammatory drugs (NSAIDs) on selected vital signs of higher plants (A) and algae s (B)

	Organism	Effects	Substances used for toxication	Year	Ref (DOI)
**A)**					
1	*Pisum sativum*	↓ protein (roots, high concentrations), changes in the anatomical structure of the root, ↓biomass and root length, ↑ membrane permeability	Naproxen (NPX)	2020	Svobodníková et al. (2020) https://doi.org/10.1016/j.chemosphere.2020.127411
2	*Oriza sativa*	↑ seedling growth at low concentrations, ↓ seedling growth at high concentration, ↑ 2,3-butanediol and ethanolamine	Ketoprofen (KET)	2020	Wang et al. (2020b) https://doi.org/10.1016/j.envpol.2020.114533
3	*Vigna unguiculata*	nutrient imbalance	IBU	2020	Wijaya et al. (2020) https://doi.org/10.3390/plants9111473
4	*Atriplex patula*, *Spinacia oleracea*, *Lactuca sativa*	oily accumulations in cell walls and vacuoles, ↓ stomatal index and stomatal pore length	DCF, IBU, and NPX	2021	Opriș et al. (2021) https://doi.org/10.1007/s42729-021-00449-5
5	*Hordeum vulgare*	↑ root length at lower concentration, ↓ root length at higher concentration	DCF, NPX	2021	Pawłowska et al. (2021) https://doi.org/10.1016/j.etap.2021.103746
6	*Pisum sativum*, *Lens culinaris*, *Vicia faba*, *and Cicer arietinum*	↓ stomatal conductance, ↓ flavonoid concentrations, ↑ 1-hexanol emission	DCF, indomethacin (IND), NPX	2022	Taschina et al. (2022) https://doi.org/10.3390/app12136326
7	*Lens culinaris*	↓ phenol concentration	DCF	2022	Taschina et al. (2022) https://doi.org/10.3390/app12136326
8	*Oriza sativa*	↓ root vitality, ↓ N metabolism enzymes,	KET	2023	Wang et al. (2023) https://doi.org/10.1016/j.envpol.2023.122485
9	*Arabidopsis thaliana*	↓ auxin transport, shortened primary roots, reduced number of lateral roots, impaired gravitropism of roots and shoots, impaired phototropism	NPX	2023	Xia et al. (2023) https://doi.org/10.1016/j.xplc.2023.100632
10	*Lactuca sativa*, *Pisum sativum*, *Allium porrum*	↓ root elongation	NPX	2024	Kummerova et al. (2024) https://doi.org/10.1007/s10646-024-02797-1
11	*Zea mays*	↑ root elongation	DCF, NPX	2024	Kummerova et al. (2024) https://doi.org/10.1007/s10646-024-02797-1
**B)**					
1	*Phaeodactylum tricornutum*	↑ lipid/protein content	Ibuprofen (IBU)	2020	Silva et al. (2020) https://doi.org/10.1016/j.marenvres.2020.105109
2	*Chlamydomonas reinhardtii*	↓ growth, cell cycle disorders	Diclofenac (DCF)	2021	Harshkova et al. (2021) https://doi.org/10.1016/j.aquatox.2020.105698
3	*Chlamydomonas reinhardtii*	↑ population density, numerous, highly electron-dense inclusions in vacuoles	IBU	2021	Moro et al. (2021) https://doi.org/10.1080/02757540.2021.1886279
4	*Chlamydomonas reinhardtii*	autophagic vacuoles	Nabumetone (NBT), Flufenamic acid (FFA)	2023	Kapuścińska et al. (2024) https://doi.org/10.1016/j.chemosphere.2023.140853
5	*Chlorella vulgaris, Desmodesmus armatus*	↓ growth, cell swelling, ↑ membrane permeability	KET	2023	Krawczyk et al. (2023) https://doi.org/10.1016/j.scitotenv.2023.165019
6	*Chlorella sorokiniana*	↑ total lipid, carbohydrate, protein content	IBU	2023	Sha’aba et al. (2023) https://doi.org/10.1007/s11356-022-22837-9
7	*Chlamydomonas reinhardtii*	membranes depolarization	IBU	2023	Seoane et al. (2023) https://doi.org/10.1016/j.aquatox.2023.106455
8	*Chlamydomonas reinhardtii*	transformation of vacuoles into larger autophagic vacuoles	DCF	2024	Harshkova et al. (2024) https://doi.org/10.7717/peerj.18005

↑ - an increase in parameter value was reported in the paper

↓ - a decrease in parameter value was reported in the paper

## Summary

**Fig. 2:**
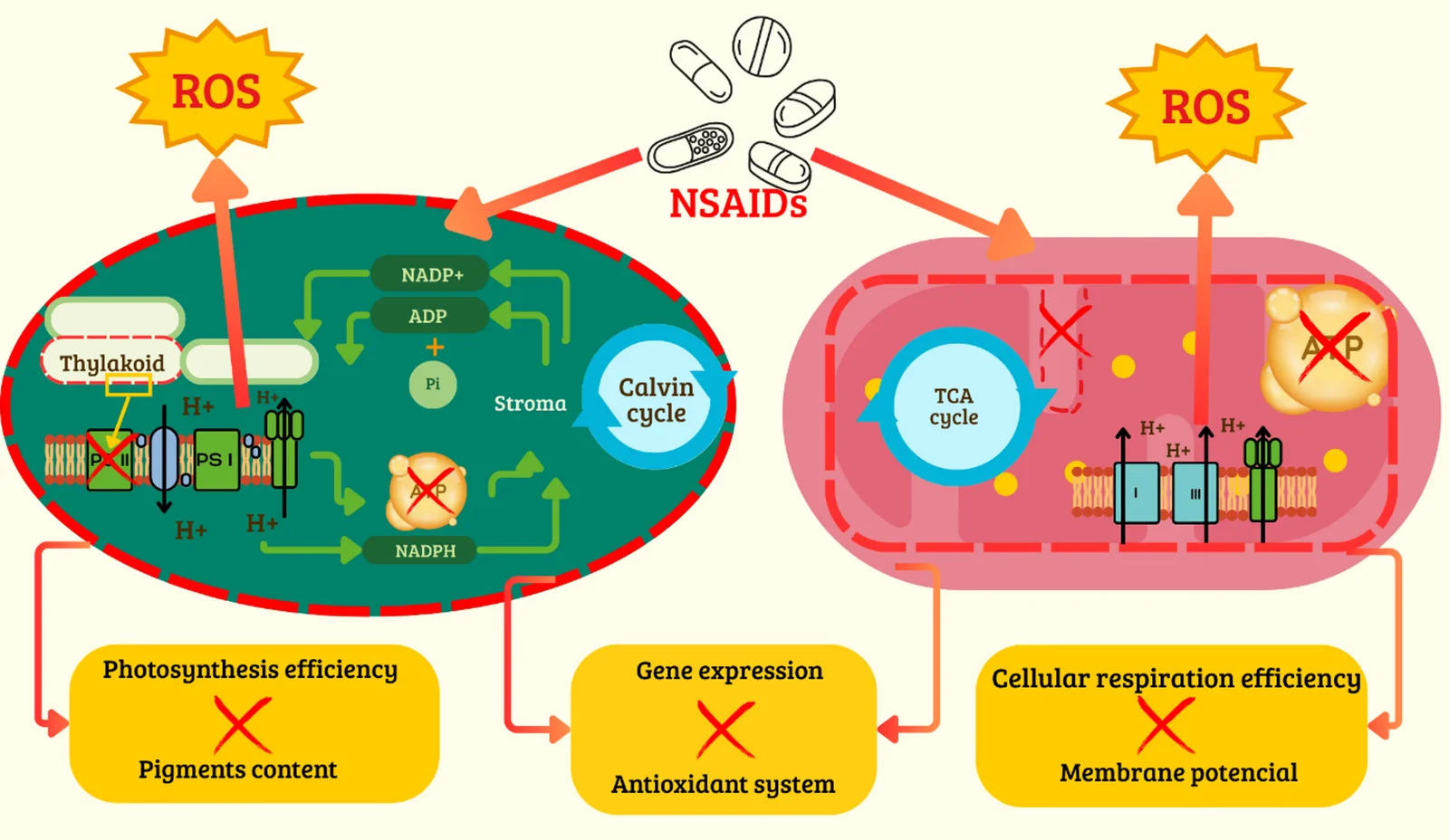
Summary of recent studies (2020–2025) on the impact of NSAIDs on photosynthesis and respiration efficiency in higher plants and algae. This figure is our own work created in Canva (Canva for Education pro license). It is used solely for educational and non‑commercial purposes in accordance with Canva’s Content License Agreement

An expanding body of literature published between 2020 and 2025 has clearly demonstrated that commonly used NSAIDs, particularly diclofenac (DCF), ibuprofen (IBU), ketoprofen (KET), and naproxen (NPX), are present in aquatic and terrestrial environments and can perturb core physiological processes in primary producers. The most consistent and well-documented effects include impairments in photosynthetic performance (reduced PSII efficiency, altered pigment composition, and chloroplast ultrastructure), disturbances of mitochondrial respiration and bioenergetics, and induction of oxidative stress with variable antioxidant responses. These functional impairments manifest in altered growth, developmental anomalies (including root architecture and stomatal function), and changes in secondary metabolism that may cascade through food webs. These conclusions are supported by multiple independent studies across microalgae and higher plants, as summarized in this review (Fig. 2). All publications collected in this review are organized into separate chapters and subsections addressing their effects on higher plants (Part A) and algae (Part B). This separated approach, both in the tables and in the text, allows us to conclude that algae often respond to NSAIDs at lower concentrations than higher plants, exhibiting rapid disruptions in photosynthesis, mitochondrial function, and redox homeostasis, likely reflecting their limited tissue-level buffering and direct cellular exposure to contaminants. Consequently, algal models provide a convenient and sensitive system for studying intracellular and subcellular mechanisms of NSAID toxicity (e.g., oxidative stress, mitochondrial dysfunction, photosynthetic electron transport) in a manner comparable to single cells of higher plants. Experiments with higher plants, in turn, allow for the assessment of whole-organism responses and developmental processes, including growth, organ differentiation, and physiological integration, that cannot be captured in unicellular algal systems.

## Research perspectives

Despite advances, significant knowledge gaps still limit the robustness of environmental risk assessment. First, much of the literature uses high, short-term exposures that do not reflect chronic, low-level environmental exposure regimes; this hinders the derivation of reliable chronic-effect concentrations and species-sensitivity distributions. Second, the identification and toxicological evaluation of NSAIDs’ transformation products remain fragmentary. Third, the interactive effects of realistic environmental mixtures (multiple pharmaceuticals, nanoparticles, microplastics, and eutrophication) are insufficiently explored. Finally, there is a shortage of mesocosm and field studies that can validate laboratory findings under ecological complexity.

To address these gaps, some important research problems should be addressed. First, chronic, concentration-response experiments across representative algal and plant taxa covering environmentally realistic ng → low µg L^− 1^ ranges with standardized endpoints should be conducted. These data are essential for the development of Species Sensitivity Distributions (SSDs) and European Environmental Quality Standards (EQS). Meanwhile, toxicant concentrations used in laboratory experiments are often substantially higher than those observed in natural environments, even in contaminated ecosystems. This approach is justified by the need to investigate targeted effects resulting from the isolated action of the studied substance, often under acute rather than chronic exposure scenarios, while excluding the interactive effects of multiple contaminants, which, in the natural environment, rarely occur individually (Daughton and Ternes 1999). It must be noticed that mixture toxicity remains insufficiently explored, despite evidence that combined NSAIDs can exert additive or synergistic effects even when individual concentrations are low. Thus, analysis of mixture and co-stressor effects in factorial mesocosm designs to estimate interaction types and ecological outcomes is necessary.

Secondly, systematic identification and quantification of major NSAIDs’ transformation products, and the examination of the toxicity of dominant transformation products in targeted assays, should become a standard component of ecotoxicity studies. The problem is important because numerous studies indicate that animals, bacteria, fungi, plants, and algae can transform NSAIDs into a variety of products with varying toxicity (Grabarczyk et al. 2020; Mulkiewicz et al. 2021; Liakh et al. 2023). The metabolism of NSAIDs in plant and algal cells is complex and varies depending on species and environmental conditions. In general, this process can be divided into three phases. Phase I mainly involves oxidation, hydrolysis, and dealkylation reactions catalyzed by cytochrome P450 enzymes. Phase II consists of conjugation with hexoses, malonic acid, or glutathione. Additionally, plants exhibit a unique phase III detoxification process, in which phase II conjugates are deposited in vacuoles or cell walls (Mulkiewicz et al. 2021). Considering the above, products of NSAID transformation constitute a large group of environmental pollutants, characterized by high variability, low predictability, and very limited characterization. Identifying the full range of NSAID biotransformation products and describing the metabolic pathways leading to their degradation is undoubtedly one of the most difficult challenges facing modern science.

The implementation of organelle-targeted studies combining time-resolved organelle probes and omics to resolve the causal sequence linking mitochondrial, chloroplast, and peroxisomal dysfunctions is another challenge to be considered in ecotoxicological studies to unravel subcellular mechanisms of pollutant toxicity beyond organism‑level endpoints. Organelle‑specific fluorescent probes targeting mitochondria, lysosomes, endoplasmic reticulum, and nuclei provide tools for real‑time visualization of stress symptoms such as oxidative stress, ion dysregulation, and membrane potential changes (Choi et al. 2021). Time‑resolved probes, particularly those compatible with fluorescence lifetime imaging microscopy (FLIM), provide quantitative and multiplexed readouts that are less affected by probe concentration or photobleaching. Such probes allow dynamic tracking of rapid toxicant‑induced events at the organelle level, capturing early molecular initiating events (Tan et al. 2025). Further, “omics” technologies, such as transcriptomics, proteomics, and metabolomics, have become essential tools for assessing complex molecular responses to environmental stressors (Li et al. 2025). Thus, integrating organelle‑resolved imaging with omics data enables linking spatially localized damage to system‑wide molecular pathways and correlating organelle phenotypes with gene and protein expression. However, there is a lack of standardized workflows for integrating time‑resolved imaging data with multi‑omics datasets in environmentally relevant models, and this is the next challenge facing ecotoxicology.

Implementing these recommendations will enhance the ecological relevance of NSAID ecotoxicology, provide regulators with robust data on chronic effects (for example, in support of proposed EQS processes), and inform remediation strategies that consider both parent compounds and their metabolites. Coupling mechanistic studies with realistic, community-level experiments and careful tracking of transformation products should be a central research aim over the coming years to reduce uncertainty in environmental risk assessment and protect primary producers that form the base of aquatic and terrestrial food webs.

## Data Availability

No datasets were generated or analysed during the current study.
